# The multilayer semantic network structure of community tensions

**DOI:** 10.3389/frma.2024.1417990

**Published:** 2024-12-02

**Authors:** Casey Randazzo, Sarah Shugars, Rachel M. Acosta, Marya Doerfel

**Affiliations:** Department of Communication, School of Communication and Information, Rutgers University, New Brunswick, NJ, United States

**Keywords:** multilayer networks, semantic network analysis, bipartite networks, disaster communication, paradoxical tensions, social media, text analysis and mining, computational social science

## Abstract

**Introduction:**

Semantic network analysis is an important tool researchers can use to untangle the knots of tension that arise as communities debate and discuss complex issues. Yet words connect not only to each other in community discourse but to larger themes or issues.

**Methods:**

In this paper, we demonstrate the use of multilayer analysis for the study of semantic networks, helping to unravel connections within and between community tensions. In examining knotted tensions that arise in the wake of disaster, this study also spotlights how climate disasters exacerbate issues like housing equity, disproportionately affecting lower-income communities. We examine discourse across eight months of online neighborhood threads about community issues in the aftermath of Hurricane Ida. We identify core tensions related to environmental sustainability, overdevelopment, neighborhood identity preservation, and economic vitality. Our within-tension analysis reveals the community's struggle with such dilemmas, while our between-tension analysis shows the interconnectedness of these issues. Our approach highlights which stakeholders are best positioned to address specific community problems.

**Results:**

The findings challenge the conventional top-down disaster response narrative, proposing a theoretically informed method for employing semantic network analysis to examine community crises. Through this work, we extend organizational communication theories of knotted tensions, offering a nuanced lens to community discourse in the face of wicked problems.

## 1 Introduction

Network scholars often grapple with the challenge of information loss when constructing bipartite projections of semantic data (Yang and González-Bailón, 2016). Preventing data loss requires the careful handling of a bipartite network's two distinct entities (Everett and Borgatti, 2013; Opsahl, 2013), which in this study are themes and their associated words. A one-mode projection of a bipartite network inherently masks information that can reveal a network's function and typology (Melamed, 2014; Vernet et al., 2014). In socio-semantic networks, this loss can include nodal attributes of concepts labeled as misinformation (Yang and González-Bailón, 2016). Everett and Borgatti (2013) propose a dual-projection approach which involves concurrently examining both one-mode projections of a bipartite network. However, this approach can neglect the hierarchical and often nested structure of language (Collins and Quillian, 1972; Rice and Danowski, 1993) which we argue is a significant limitation when applied to semantic networks. We find that traditional bipartite networks obscure the distinctiveness of themes, preventing scholars from examining discourse similarity using correlation coefficients. This limitation has the potential to lead to misinterpretations in how semantic themes interact and influence each other. In response, we advocate for a multilayer approach for the analysis of bipartite semantic networks. In this paper, our network maintains two distinct modes. The first mode represents a set of thematic layers and the second mode reflects a set of words associated with those layers. This approach enables us to observe how words interact both within (intra-layer) and between (inter-layer) themes. In doing so, we extend prior work on multilayer networks which is often limited to studies using socio-semantic or social network data (Kleinnijenhuis and De Nooy, 2013; Knoke et al., 2021). We argue that a multilayer approach better reflects the structure of discourse in which words serve to build and connect themes (Woelfel et al., 1980).

To demonstrate the multilayer approach, we turn to the concept of knotted tensions (Sheep et al., 2017) which can be used to examine how contradictions interconnect in public discourse (Fairhurst and Putnam, 2023). Particularly, we focus on an online neighborhood Facebook Group following Hurricane Ida. Disasters are complex and present a plethora of challenges to individuals, communities, and organizations (Doerfel et al., 2022; Kim et al., 2022; Spialek and Houston, 2018a,b). Community reactions to disaster-related issues can be found on social media platforms like Facebook or Nextdoor (Benedict, 2022; Bird et al., 2012; Bortree and Seltzer, 2009; Chewning et al., 2013; Liu et al., 2018; Stephens et al., 2020). This context often makes the existence of tensions salient, emerging from oppositional opinions or disagreements on potential courses of action (Fairhurst and Putnam, 2023; Heath and Frey, 2004; Koschmann and Isbell, 2009; Lange, 2003; Sundaramurthy and Lewis, 2003; Zoller, 2000). Tensions are different from topics or themes, which aggregate large bodies of text into broadly summarized titles to succinctly reflect a complex set of text data. Instead, tensions refer to the “push-pull dilemmas among choices” (Fairhurst and Putnam, 2014, p. 279) that result from competing available options. These tensions are often separated into two poles and managed through individuals choosing or prioritizing one pole over another as new situations arise (Smith and Lewis, 2011). However, this does not necessarily resolve tensions since they exist in multiples, meaning they interconnect and persist over time as new options emerge for individuals to choose between (Fairhurst and Putnam, 2024). Using qualitative thematic analysis, we identified five community tensions: (1) Grassroots Organizing vs. Bureaucratic Expectations; (2) Tenant Rights vs. Landlord Obligations; (3) Environmental Sustainability vs. Overdevelopment; (4) Neighborhood Preservation vs. Economic Progress; and (5) Progressive vs. Conservative Values. We use these tensions to define our bipartite network with the first mode representing the thematic layers and the second reflecting the words associated with each layer.

We utilized both Python and R to construct the networks, which include the multilayer network and the traditional bipartite network for comparison. We then calculated centrality metrics within thematic layers and interlayer correlations across layers. Findings demonstrate how layers interconnect, reflecting the semantic structure of knotted tensions (i.e., multiple interconnected tensions; Fairhurst and Putnam, 2023; Raja et al., 2022; Sheep et al., 2017). Between-layer analyses then illustrate the interconnectedness of these tensions, demonstrating how tensions knot within community dialogue. In the context of disaster research, these findings flip the usual post-disaster script where agencies bring top-down agendas to response and recovery. More broadly, we argue that multilayer representations of semantic networks are particularly well-suited for uncovering discursive interconnections within public spheres.

Theoretically, we extend organizational communication work on knotted tensions (e.g., Fairhurst and Putnam, 2014; Sheep et al., 2017) by providing a methodological framework to quantify these interconnections. Our approach moves beyond mere identification of tensions and instead offers a way to operationalize their interconnectedness, enabling scholars to quantify how tensions knot and coalesce across community discourse. Practically, this method can help practitioners, such as emergency response teams, policymakers, and community organizers, to recognize the layered nature of tensions, as opposed to treating them as isolated issues. This understanding can inform intervention strategies and help agencies engage with communities more effectively during disaster recovery. By identifying overlapping tensions, practitioners can tackle multiple issues at once, streamlining recovery efforts and minimizing the need for separate resource allocation.

## 2 Review of literature

### 2.1 Tensions in publics

Publics are communicative spaces where actors discuss and debate matters of common concern (Habermas, 1989; Dewey, 1927; Stoltenberg, 2024). Social media has provided particularly rich access to publics, as such discourse increasingly plays out—and is recorded—in online settings. Issues that garner attention in publics are often surrounded by tension (Putnam et al., 2016), particularly when they involve oppositional outcomes (Fairhurst and Putnam, 2023). Although publics often enable sensemaking (Habermas, 1996; Mansbridge, 1992; Mercier and Landemore, 2012), members can still struggle to define tensions surrounding community issues (Miller and Riechert, 2001). Actors, therefore, make decisions by managing or responding to tension (Woo, 2019). However, tensions are often left unresolved (Smith and Lewis, 2011) as they are impervious to resolution (Schad et al., 2016). Individuals often use different communicative strategies when navigating tensions (Fairhurst and Putnam, 2024; Smith and Lewis, 2011). For example, the “both-and” strategy is a dialectical approach where tensional poles are seen as interdependent as opposed to mutually exclusive (Berti and Simpson, 2021). In the context of organizing, such strategies can help communities navigate stakeholder relationships (Gordon and Lopez, 2019; Zoller, 2000) and address local needs (Cooper, 2021; Koschmann and Isbell, 2009).

Tensions can persist through text (Putnam et al., 2016; Smith and Lewis, 2011), making semantic network analysis an ideal method for exploring tensions in social media discourse (Eddington, 2020; Jarvis and Eddington, 2021). In their analysis of discussions on the subreddit r/TheRedPill, Jarvis and Eddington (2021) used semantic network analysis to identify tensions related to subordination and empowerment, revealing how they contribute to anti-feminist ideologies.^1^ Such studies, however, have not fully utilized semantic network analysis to uncover the interconnections within tensional knots, defined as multiple interconnected tensions (Fairhurst and Putnam, 2023; Raja et al., 2022). In organizing contexts, tensions “develop in complexity” as contradictory opposites evolve, persist, and interconnect (Fairhurst and Putnam, 2014, p. 279) forming a complex configuration that resembles a knot (Sheep et al., 2017). Using prisms as a metaphor, Sheep et al. (2017) illustrated how knotted tensions can have implications for action. Similar to how a prism disperses and intensifies light, the structure of tensional knots can scatter a community's focus and energy, making it difficult to find a clear path forward (Sheep et al., 2017).^2^ Collectively, such work underscores the importance of investigating the networked structure of community tensions.

### 2.2 Social media discourse

Social media conversations, which reflect written records of community discussion (Shugars, 2019), are a ripe place for the examination of tensional knots (Eddington, 2020; Jarvis and Eddington, 2021). Language—whether in words, sentences, or groups of utterances—constructs social contexts (Gallagher et al., 2018; Howarth, 2000; Lawrence et al., 1999; Schiffrin, 2006) and can act as a resource upon which one draws knowledge (Hardy et al., 2005; Habermas, 1989). As prior work explains, one's linguistic choices can reflect deep-seated meanings (Jørgensen and Phillips, 2002; Potter and Wetherell, 1987), serving as windows into the function of discourse (Doerfel and Barnett, 1999; Doerfel et al., 2013; Eisenberg, 2007; Giddens, 1979, 1987). Individuals often use language to explain their ideas and actions (Doolin, 2003; Shugars and González-Bailón, 2023) in online spaces (Liu et al., 2018; Yuan et al., 2013). Here, social media discourse functions as more than a mechanism for information exchange or diffusion (Beauchamp, 2020; Matassi and Boczkowski, 2023) but as a discursive space for community discussions and cross-cultural dialogue (Papacharissi, 2009; Randazzo and Ammari, 2023).

This study focuses on community discourse in online neighborhood groups, which are digital interest or topic communities tied to geographic regions (Hampton and Wellman, 2003; Wellman et al., 2001). Hosted on social media platforms like Facebook or Nextdoor (Lambright, 2019; Lee and Ahn, 2023), neighborhood groups often contain informal conversations about public life (Benedict, 2022; Carpini et al., 2004; La Due Lake and Huckfeldt, 1998; Kim et al., 1999; Kurwa, 2019; Weatherford, 1982). In the aftermath of a weather disaster, neighborhood groups can act as hubs for preparation and management (Bird et al., 2012; Bortree and Seltzer, 2009; Chewning et al., 2013; Stephens et al., 2020). Disasters are traumatic, requiring the need for collective sensemaking through public discourse (Alexander, 2004; Eyerman, 2004; Rimé, 2020). Such community discourse is often riddled with tensions as communities discuss paths toward recovery (Dryzek, 2005; Mercier and Landemore, 2012). Discourse can allow communities facing a disaster to identify and frame tensions in a productive way to overcome obstacles (Driskill et al., 2012). By examining online neighborhood discourse, we seek to shed light on the dynamics of discursive organizing during times of crisis.

### 2.3 Semantic networks

Semantic network analysis has its roots in the assumption that language is structured hierarchically (Collins and Quillian, 1972; Osgood et al., 1978) where interconnected words contribute to the meanings of phrases (McGee, 1980). By drawing connections between related words or concepts, we come to understand the social structure of language and meaning (Woelfel et al., 1980). Semantic network analysis involves applying “network analytic techniques on paired associations” (Doerfel, 1998, p. 16) which enable researchers to investigate and uncover shared meanings from connected words or concepts (Shugars and González-Bailón, 2023). For example, Yuan et al. (2013) utilized semantic network analysis to uncover how Chinese Internet users defined the meaning of privacy.

Carley and Kaufer (1993) describe the links between concepts as representative of varying levels of social (dis)agreements in the form of “provocative text that contains debates, tensions, contradictions, biases, explicit, or implicit agendas” (Segev, 2021, p. 16). Semantic network analysis serves as a tool for interrogating the shared narratives, language, and codes of communities (Danowski, 1993; Doerfel, 1999; Shugars and González-Bailón, 2023).

Network scholars have increasingly recognized the potential of semantic networks to uncover patterns in social media discourse (Featherstone et al., 2020a). Danowski et al. (2024) provide a significant contribution by employing a cascaded semantic fractionation approach, which involves removing high betweenness terms to identify more coherent subgroups within semantic networks. Danowski et al.'s (2024) approach helps untangle complex semantic structures while avoiding information loss. Similarly, Featherstone et al. (2020b) have explored public discourse around controversial topics such as childhood vaccination, revealing how semantic networks can provide insight into community opinion clusters.

By building on these foundations and applying a multilayer framework, this paper contributes to the growing body of work that uses computational methods to map and analyze discursive structures within social media contexts (Hilbert et al., 2019). Specifically, we use partitions, which in this study reflects high-level themes, to infer meaning from clustered concepts (Arasaratnam and Doerfel, 2005; Doerfel and Marsh, 2003; Yuan et al., 2013), relative centrality to assess which concepts constitute community tensions, and density (Doerfel and Fitzgerald, 2004) to indicate how tensions are layered within the larger neighborhood network. We argue that a multilayer approach brings richer insight into the discursive dynamics captured by semantic networks. We accomplish this by examining linguistic connections both within and across themes (layers). That is, our approach allows for analyzing both how words are used to articulate themes (within layer analysis) and how concepts interconnect with each other (between layer analysis). In doing so, we respond to ongoing calls to extend semantic network analysis beyond traditional one-mode or bipartite structures and to better account for the complexity of discourse in public spheres (Featherstone et al., 2020a; Hilbert et al., 2019). Thus, we examine the following research questions:

**RQ1:** What are the community tensions present in multiple layers of online neighborhood discourse?
**RQ2:** How do thematic layers of tensions interconnect across online neighborhood discourse?

## 3 Materials and methods

This paper adopts a computational social science approach, which is often described as an inductive process that complements traditional, qualitative methods (Ylä-Anttila et al., 2022). Given the implicit network structure of “tensional knots,” we focus particularly on using semantic network analysis to understand and unravel such complex, interconnected structures.

### 3.1 Data collection

Our dataset includes 1,489 posts made in a neighborhood Facebook group from September 2021 to March 2022. This community group serves a densely populated borough in the greater New York Metropolitan Region that was devastated by the Category 4 Hurricane Ida on August 26, 2021. Ida caused an estimated 65 billion dollars in damages to this community, displacing many long-term residents, both homeowners and renters (McKinley et al., 2021; Wood, 2022). Data were collected in 2022 through Meta's Crowdtangle API (Meta, 2022) and was approved by the Rutgers University Institutional Review Board IRB. We anonymized the dataset by obscuring user, street, building, and stakeholder names (e.g., mayor, developer, governor) mentioned in posts. Names were replaced with informative placeholders such as “developer1” or “organizer1” to allow context to be reflected in our qualitative analysis while ensuring anonymity. The specific tags used for each entity were informed by one author's deep knowledge of the community context as well as our qualitative review of anonymized texts. For example, we ultimately gave the most tagged username the identifier of “organizer1” to reflect their efforts organizing donations and collective action.

### 3.2 Thematic analysis

To answer RQ1, we conducted thematic analysis of conversation threads. Each thread represented an initial post followed by replies. The conversation trees have a maximum depth of 2, as replies can receive replies of their own, but Facebook does not allow further nesting. We considered each thread to be its own coherent conversation and conducted our analysis at the thread level. This is because replies made to an initiating post, or even further nested replies, typically engaged with each other and reflected a coherent conversational experience. In total, our dataset consists of 354 threads, with an average length of 24 comments.

We utilized Braun and Clarke's (2006) guidelines on qualitative coding to uncover thematic patterns and to surface the tensions reflected in these threads. This involved us first immersing ourselves in the data, reading and re-reading conversation threads to become familiar with the depth and breadth of content. This first phase helped to identify preliminary codes that could point to underlying tensions. After immersing ourselves in the data, two of the authors meticulously annotated the dataset to identify common themes. We then iteratively refined and modified the identified themes over multiple discussions to accurately capture the essence of the dataset.

Inter-rater reliability was not needed for this study due to the iterative nature of our analysis. We allowed the themes to emerge organically, using a bottom-up approach (Braun and Clarke, 2006) to identify the tensions present in the conversation threads. Our goal was not to quantify agreement across coders, but to collectively identify and refine the themes that best captured tensions. Once the themes were established, we used prior work to help contextualize the titles and ensure alignment with existing research without altering the meaning of the themes.

This work resulted in five distinct tensions: Progressive vs. Conservative Values, Neighborhood Preservation vs. Economic Progress, Environmental Sustainability vs. Overdevelopment, Tenant Rights vs. Landlord Obligations, and Grassroots Organizing vs. Bureaucratic Expectations. Each thread was then identified as relating to one of these tensions. Additionally, while all data was collected from a public group, we followed the recommendations of Bruckman (2002) and described quotes to ensure that comments were undiscoverable. The findings from the thematic analysis laid the groundwork for generating network layers reflective of each tension.

### 3.3 Topic modeling

**Figure 1:**
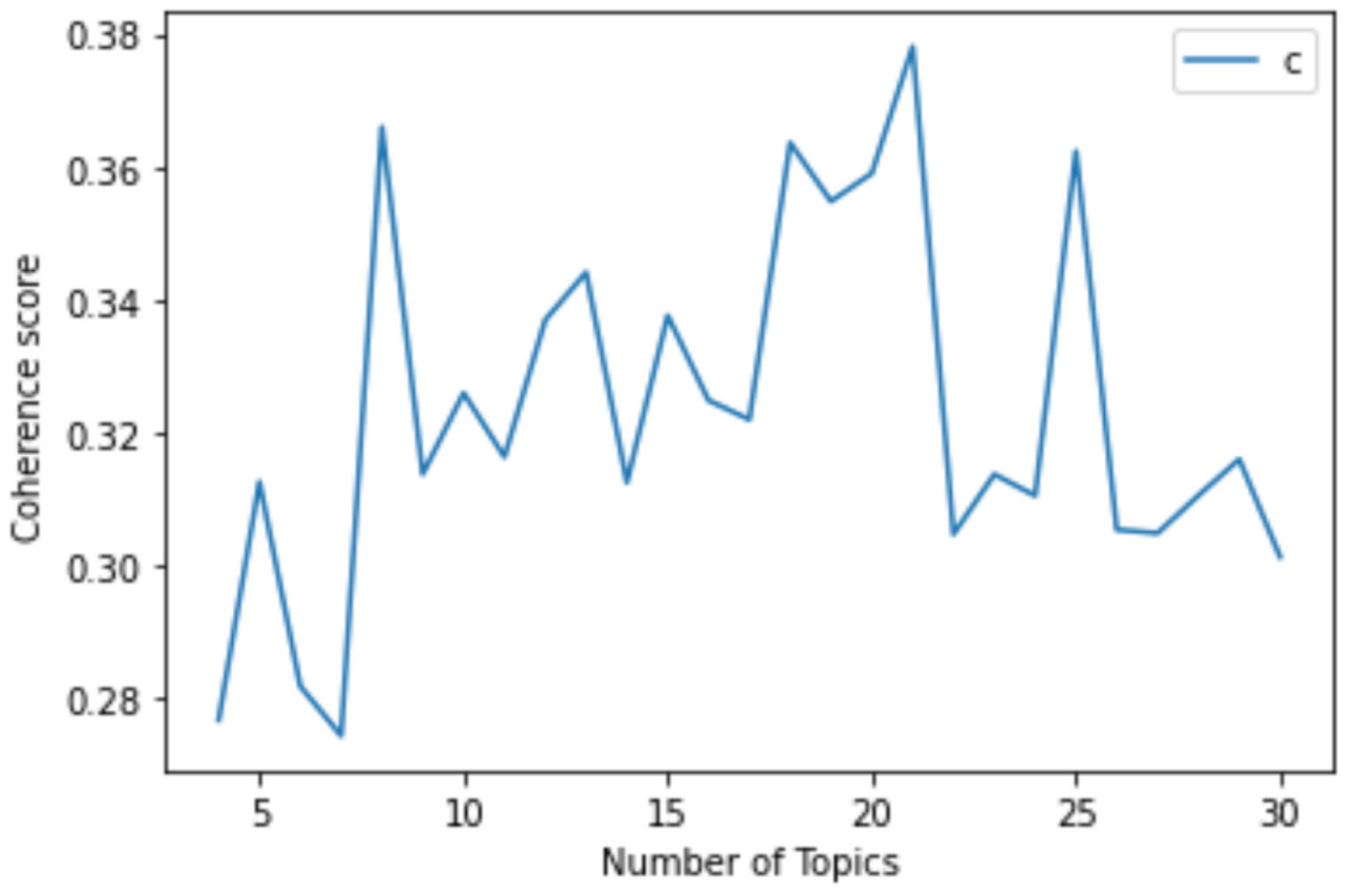
Coherence of Topic Models: This graph displays the variation in coherence scores across different topic models.

We further validated our qualitative analysis using topic modeling, a computational approach, which surfaces the latent dimensions reflected in a collection of texts. This approach allowed us to both confirm that our five qualitatively identified themes are linguistically distinct across our corpus and to identify specific “topics” which comprise each of these themes. For this task, we used Latent Dirichlet Allocation (LDA), a common topic modeling technique that is scalable to large datasets (Blei, 2012; Blei et al., 2003; Maier et al., 2018). As compared to newer methods (e.g., BERTopic, Top2Vec), LDA has been found to produce less interpretable or overly generalized topics (Egger and Yu, 2022). These potential issues were less of a concern for this study as we were not using LDA to discover insights but instead to confirm findings from the qualitative analysis. We determined the ideal number of topics based on coherence scores, which are a metric for assessing topic models by calculating the semantic similarity of high-probability words inside each topic. We generated the topic models using Gensim's LDA implementation in Python (Röder et al., 2015). We then compared the coherence scores (Figure 1) of 5 topics (~0.31), 21 topics (~0.38), and 25 topics (~0.36). After reviewing the keywords for each, we chose 21 Topics as it produced the most coherent outputs. However, two of the topics (Topic 18 and Topic 19) were ultimately deemed uninterpretable through qualitative assessment, and therefore, were excluded from further analysis.

**Figure 2:**
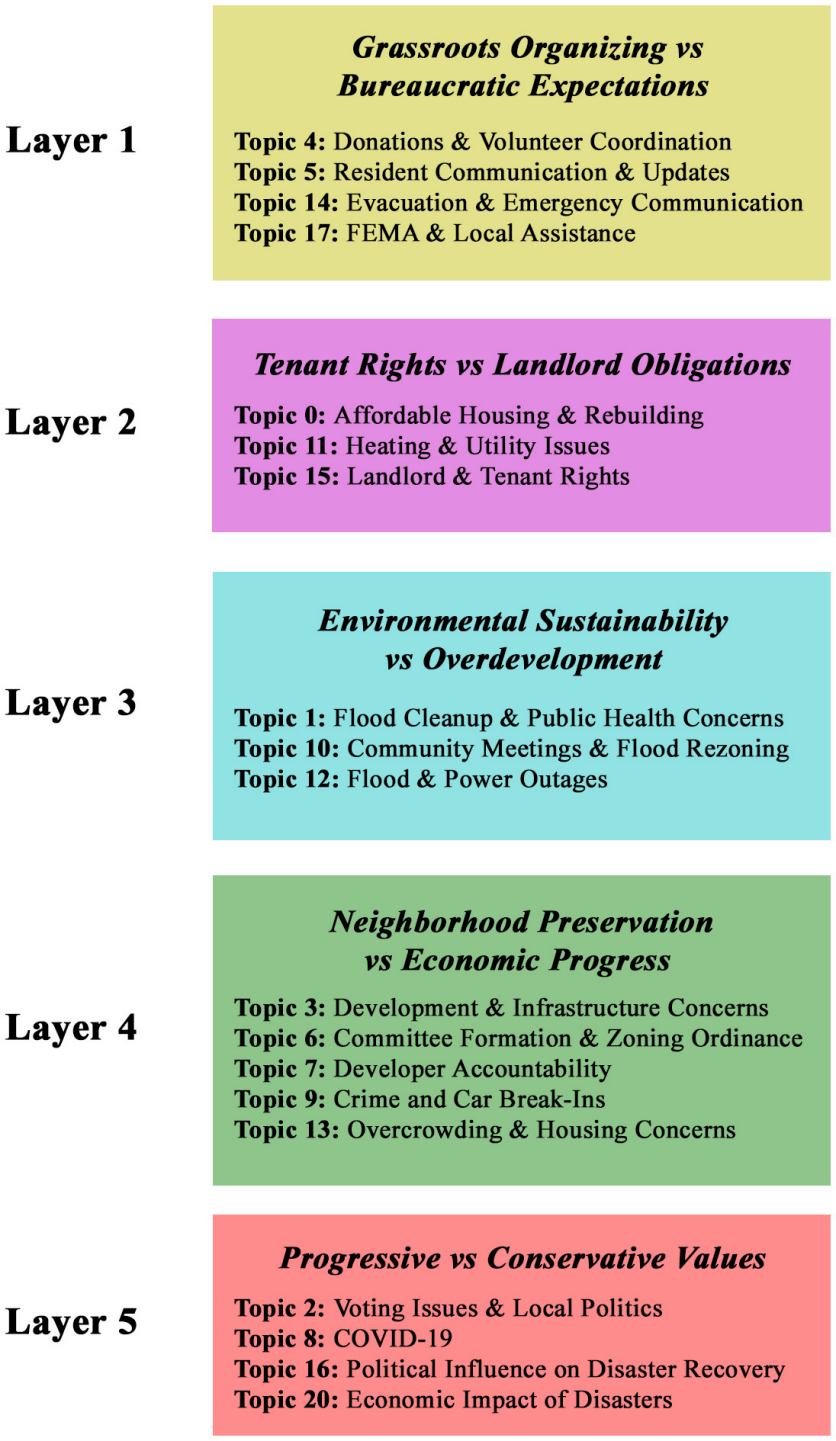
Thematic grouping of topics: We conducted qualitative coding on a sample of comments that were in the 98th percentile of relevance to each topic. These topics were then grouped under the five overarching tensional layers.

We generated an Intertopic Distance Map (Sievert and Shirley, 2014) that uses multidimensional scaling to show the relationships between subjects in a two-dimensional environment. The size of the circle denotes the relative frequency of each topic in the dataset, and each circle represents a topic (Appendix). More popular topics, such as Topic 1, are reflected as larger circles. The lengths between the circles show how similar the topics are to one another; distant topics use different language, while closer topics have more overlapping words. The right panel in Appendix shows the Top-30 Most Relevant Terms for Topic 1, accounting for 30.3% of the tokens in the dataset. In the image, the blue bars represent the overall term frequency in the entire corpus and the red bars show the estimated frequency of each term within the selected topic. The Intertopic Distance Map allows us to view which words are both frequent in the dataset and highly relevant to Topic 1. However, the keywords in Appendix are limited in contextualizing the topic. Therefore, we sampled comments that were in the 98th percentile of relevance to each topic. We then conducted qualitative coding on the sampled comments, allowing us to generate meaningful labels for each topic. These topics were then grouped under the five overarching tension layers (Figure 2). By linking the results of topic modeling to these tensions, we were able to systematically map the discourse into distinct thematic layers that reflect the structural and semantic patterns of the conversation threads.

### 3.4 Semantic network analysis

This paper uses semantic network analysis due to its usefulness in uncovering the structure of online conversations (Paulus and Wise, 2019; Shugars and González-Bailón, 2023). We constructed seven networks in total: one multilayer network with within-layer (word-to-word) and between-layer edges (tension-to-tension via shared words), five semantic networks representing the layers of the multilayer network, and one bipartite network projection (tension-to-tension via shared words) for comparison. We began by preprocessing the text in each post, making all words lowercase, as well as removing punctuation and stopwords. In addition to tidytext's stopword list in R, we added custom stopwords that were particularly common in our data such as “comment” and “post.” Our qualitative analysis revealed that these words often referred to the medium of Facebook itself and did not provide meaningful insight into the content of the conversation.

Using the clean text, we then constructed co-occurrence networks for each tension. As Danowski (1993) explains, co-occurrences enable researchers to investigate meaning embedded in text. Words were considered connected if they co-occurred within the same thread. In other words, we took all threads identified as related to a given tension and considered each word in those threads as a node in that tension's semantic network. We then defined the weight of connection between two words as the number of times those words co-occurred within the same thread. For example, if the words “tenant” and “heat” both appeared 5 times within a single thread, those nodes would have an edge of weight 5 connecting them. These weights were additive across threads related to the same tension, so if a second thread also had a co-occurrence of the words “tenant” and “heat,” the edge weight in the associated tension network would increase to 6.

We examined co-occurrence at the thread level rather than at the comment level because we were interested in getting a broad picture of what words and concepts were connected to each tension. That is, rather than comparing comments made in back-and-forth debate, our analysis better quantifies how each tension itself is reflected in discourse. Previous work has found that aggregating textual data to the thread-level allows researchers to respect the integrity of such interactions in a way that is missed when isolating sentences or comments (Herring, 2004), an approach that overemphasizes incidental word pairings. Furthermore, analyzing semantics on the thread-level acknowledges social media discourse as a dynamic interplay of communicative actions which include how users react, respond, and contribute to posts.

To answer RQ2, we constructed both a multilayer and a bipartite network projection network to assess the interconnections between tensions. In the traditional bipartite network, each word that appears within a tension's network is considered linked to that tension. This approach reveals the word co-occurrence between, rather than within tensions. For example, if the words “tenant” and “heat” are connected to Tension 1 and the words “tenant” and “developer” are connected to Tension 2, then the projection of the bipartite network onto the tension nodes will show the high-level linguistic interconnections between those tensions. These between-tension connections are weighted by the number of unique words shared by two tensions.

The multilayer network provides a detailed view of the connections between and within tensions (Artime, 2022). As Knoke et al. (2021) explain, multilayer networks “preserve all relational ties rather than erasing some information through projections which collapse data across modes” (p. 2). Multilayer networks can be used to visualize multiple types of connections between the same nodes (Barnett et al., 2013; De Domenico et al., 2015). The layers within these networks can represent different time periods (e.g., disaster phases) or interactions (e.g., email, meetings), which collectively form a more complete picture of complex systems (Boccaletti et al., 2014). In this study, each layer within the multilayer network represents one of the five tensions uncovered through the thematic analysis. Intra-layer edges (i.e., links between words within a single layer; Kinsley et al., 2020) represent the co-occurrence of words, illustrating how terms cluster around specific topics. Inter-layer edges are connections between different layers. The same node that appears in any two layers forms a coupling edge (Kivela et al., 2014), bridging multiple themes.

### 3.5 Network measures

#### 3.5.1 Centrality measures

To analyze our semantic networks, we considered a range of network statistics. Specifically, for our tension-level semantic networks, we examined degree, betweenness, eigenvector, and closeness centralities, along with clustering coefficients (Scott, 2017; Wasserman and Faust, 1994). Degree centrality, which measures the total number of times a word co-occurs with others, allows us to identify concepts with a high number of connections within the network (Segev, 2021). Betweenness centrality measures how frequently a node falls between other nodes in the network (Xu, 2020), filling structural holes (Burt, 1992). While closeness centrality measures how closely, on average, a node is connected to all other nodes. Eigenvector centrality identifies influential nodes based on their connections to other important nodes within the network, helping to uncover central themes or concepts (Bonacich, 1987). The clustering coefficient measures the extent to which nodes tend to cluster together, which can signify a thematic cluster or a closely knit group of concepts reminiscent of dense, small-world networks (Watts and Strogatz, 1998).

#### 3.5.2 Interlayer correlations

To examine connections between tensions, we calculated interlayer correlations through the multilevel network (Dickison et al., 2016). We operationalized interconnections between tensions as Berlingerio et al.'s (2013) layer correlations which adapts the Jaccard correlation coefficient for multilayer networks. Jaccard correlation coefficients are traditionally used to measure the similarity between two sets by dividing the size of the intersection of the sets by the size of their union. In multilayer networks, layer correlation represents the similarity in the presence of edges among the same actors across different layers (Berlingerio et al., 2013). To compute this metric, we calculated Jaccard Coefficients (size of the intersection and union) between a set of unique edges for two given tensions.

## 4 Results

### 4.1 Layers of community tensions

In the following section, we provide an in-depth analysis of the tension layers (RQ1) within the multilayer network and the topics within each layer. We then discuss the unique interconnections that occur between layers of tensions (RQ2). Our findings revealed five layers that reflect social media discourse following disaster: (1) Grassroots Organizing vs. Bureaucratic Expectations, (2) Tenant Rights vs. Landlord Obligations, (3) Environmental Sustainability vs. Overdevelopment, (4) Neighborhood Preservation vs. Economic Progress, and (5) Progressive vs. Conservative Values.

We found 19 topics within these tensions (Figure 2). Topics under Layer 1, Organizing vs. Bureaucratic Expectations, included donations and volunteer coordination (Topic 4), resident communication and updates (Topic 5), evacuation and emergency communication (Topic 14), and FEMA and local assistance (Topic 17). Topics under Layer 2, Tenant Rights vs. Landlord Obligations, included affordable housing and rebuilding (Topic 0), heating and utility issues (Topic 11), and landlord and tenant rights (Topic 15). Layer 3, Environmental Sustainability vs. Overdevelopment included flood cleanup and public health concerns (Topic 1), community meetings and flood rezoning (Topic 10), and flood and power outages (Topic 12). Layer 4, Neighborhood Preservation vs. Economic Progress, included development and infrastructure concerns (Topic 3), committee formation and zoning ordinance (Topic 6), developer accountability (Topic 7), crime and car break-ins (Topic 9), and overcrowding and housing concerns (Topic 13). Layer 5, Progressive vs. Conservative Values included voting issues and local politics (Topic 2), COVID-19 (Topic 8), political influence on disaster recovery (Topic 16), and the economic impact of disasters (Topic 20). As mentioned in the methods section, we omitted Topic 18 and Topic 19 from the model as they were uninterpretable.

#### 4.1.1 Layer 1: grassroots organizing vs. bureaucratic expectations

Layer 1 reflected a tension between grassroots organizing to address community needs and the expectations and responsibility of local government officials. Residents expressed frustration with the lack of preparedness by officials for Hurricane Ida (Topic 5) and felt poor communication resulted in the destruction of emergency vehicles needed for rescue efforts (Topic 14). Network metrics for Layer 1 revealed high eigenvector and betweenness scores for both “mayor” (eigenvector: ~0.94; betweenness: 5,907) and “council” (eigenvector: ~0.97; betweenness: 626), reflecting the influence of such leadership positions within the community. “Agree” held the highest betweenness centrality (8,160) and eigenvector (1.0) scores, which can be evidence of deliberative discussion as the term can reflect points of contention or consensus within the discourse.

We observed significant centrality results for “flood” (betweenness: 4,261; closeness: 0.000465) and “water” (betweenness: 3,288; closeness: 0.000536) which suggests that these words formed a sub-network of discussion around water management issues and the town's flood response. The words “tenants,” “child,” and “care” share a strong connection to “house,” which held the highest betweenness centrality (~9,219). This result echoed the decision-making processes around organizing local assistance (Topic 17). Community members appeared to prioritize donations and housing arrangements for displaced users with children (Topic 4). The relatively lower clustering coefficient (0.253) for “house” suggested that discussions about housing are widespread, involving diverse parts of the network as opposed to being confined to a tightly knit cluster of words.

#### 4.1.2 Layer 2: tenant rights vs. landlord obligations

**Figure 3:**
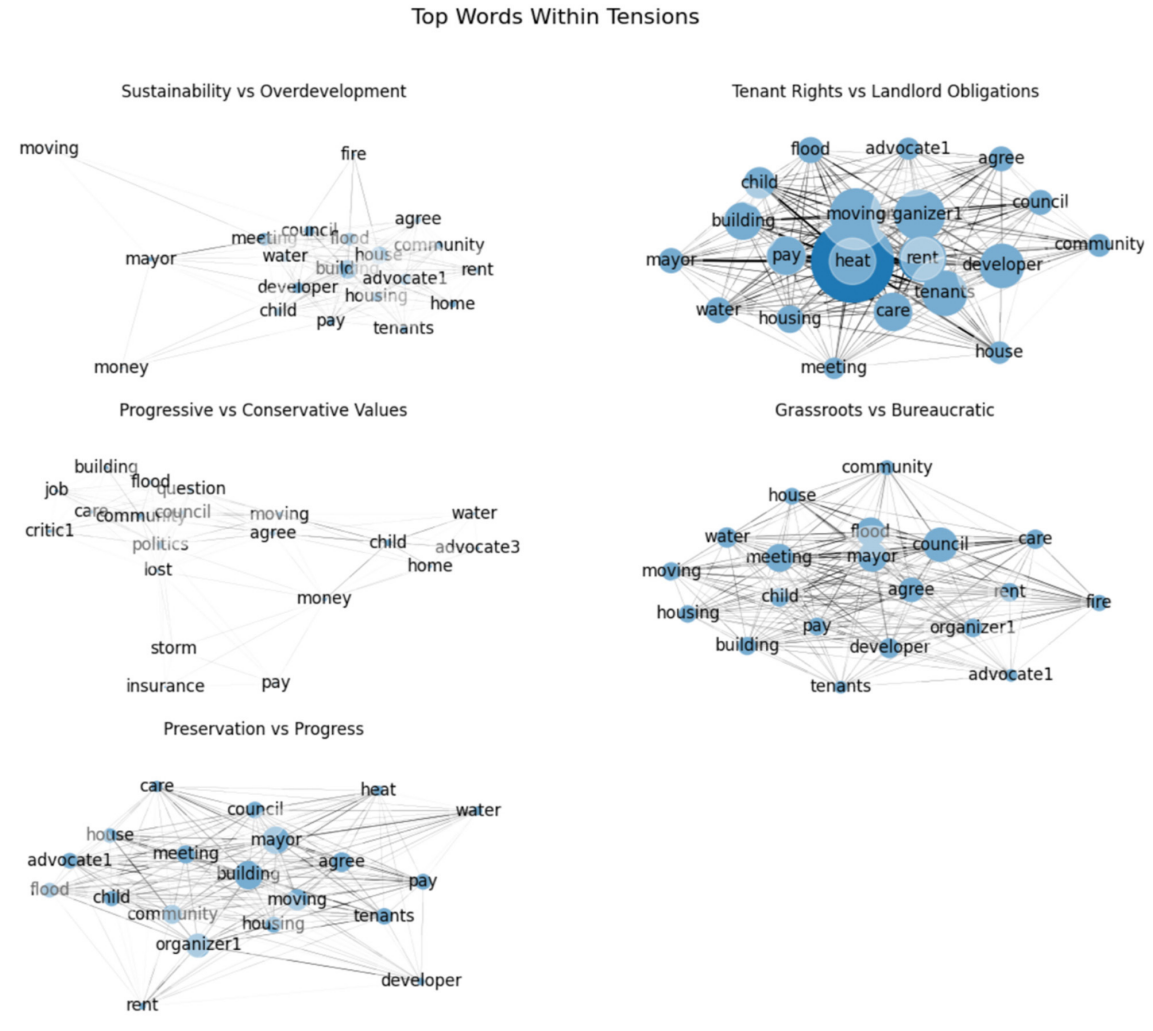
Tension Networks: This visual reflects the 20 most frequent words within each tension network. Node size is equivalent to frequency of term.

Layer 2 revealed the power struggle between individuals seeking improved living conditions vs. the obligations of their landlords. Users personally experiencing these conditions described the deterioration of their apartment buildings (Topic 11). Replies included guidance on safely using space heaters, contacting media outlets with complaints, beginning a letter writing campaign to local municipality leaders, and hiring tenancy law attorneys (Topic 15). Community members encouraged others to attend council meetings and help advocate for affordable housing (Topic 0). This analysis demonstrated the duality of tensions as members encouraged others to leverage their knowledge of tenant rights and attend town hall meetings. Network metrics revealed “heat” to be a significant concern for users. The high clustering coefficient (~0.602) and betweenness centrality (3,062) score for “heat” reflected tightly knit discussions about heating issues and the term's role as a bridge to named stakeholders (“organizer1,” “council,” “mayor,” “developer”). Of the named stakeholders, “organizer1” had the highest betweenness centrality (7,392) and “mayor” among the highest clustering coefficients (~0.844). The connections between “heat” and those named coupled with network metrics reflected the power and responsibility of such positions relative to unsafe housing practices. Such evidence aligned with social network analysis results as “meeting” had one of the highest clustering coefficients (0.454) for this network (Figure 3).

#### 4.1.3 Layer 3: environmental sustainability vs. overdevelopment

Layer 3 focused on the environmental consequences of overdevelopment. In this layer, community members discussed the consequences of rezoning proposals that would allow for the construction of “green housing” in flood zones (Topic 10). Members also suggested redesigning uninhabitable apartment complexes with parking garages on the first floor as a flood mitigation strategy (Topic 12). Semantic network analysis revealed the prominence of terms that highlight the consequences of disasters. For instance, “water” (eigenvector: ~0.996; betweenness: 3,100), “building” (eigenvector: 1.0; betweenness: 2,736), and “flood” (eigenvector: ~0.997; betweenness: 2,791) shared high centrality scores. These results reflect conversations about water damage and power outages caused by Hurricane Ida (Topic 1). Findings demonstrated the community's concerns about the consequences of overdevelopment in flood zones and frustration over the lack of qualified professionals on the zoning board committee (Topic 10). Terms like “mayor” (~4,045) and “council” (~1,769) also held high betweenness centrality, speaking to the community's recognition of local governance being a pathway to address local issues. These findings suggested that governance and decision-making processes are significant points of discussion and potentially lead to new avenues for collective action.

#### 4.1.4 Layer 4: neighborhood preservation vs. economic progress

Layer 4 reflected community members' discussions about preserving neighborhood identity vs. experiencing economic growth. Semantic analysis results for Layer 4 revealed “building” as a focal node with high degree centrality (915) and eigenvector centrality (1.0). Hurricane Ida caused infrastructure damage, providing the opportunity to either restore or replace uninhabitable low-income apartment buildings with luxury living facilities (Topic 6). Community members rallied others to attend town hall meetings and encouraged the mayor to hold the developer accountable and address gentrification (Topic 7). “Housing” (degree: 887; eigenvector: ~0.998) and “community” (degree: 870; eigenvector: ~0.997) also showed high degree and eigenvector centrality scores. These measures pointed to housing issues such as overcrowding and the broader socio-economic fabric of the neighborhood being a pressing concern for community members (Topic 13). Despite playing important roles, “housing” and “community” occupied different positions in the network.

“Community” had a higher betweenness centrality (~1,102) as compared to “housing” (~278), indicating that “community” functioned as more of a connector between different parts of community discourse. Users also blamed local government for the change in neighborhood identity, which aligned with semantic network results. The high clustering coefficient (1.0) and low betweenness centrality (0.0) for “mayor” implied that such conversations were highly focused on critiques and endorsements of the mayor's actions (or inactions) toward neighborhood preservation. Others connected neighborhood preservation to “child” which held a high degree centrality (870) and eigenvector centrality scores (0.997). For example, individuals pointed to a lack of activities for children as evidence of the town prioritizing the construction of luxury apartments (Topic 3) over amenities for families. The betweenness centrality of “child” (237), while not as high as other terms, still suggested relation to other observed concerns like the overcrowding of public schools and the increased cost of living.

#### 4.1.5 Layer 5: progressive vs. conservative values

Layer 5 involved politically charged conversations (Topic 2), which was also evidenced by the centrality scores of terms like “politics” (eigenvector: ~0.677) and “question,” holding the highest betweenness centrality score (~4,570). The presence and frequency of terms like “money” and “job” underscored financial pressures, potentially resulting from economic ramifications from COVID-19 (Topic 8) and/or Hurricane Ida (Topic 20). “Money” had a high betweenness centrality (~2,424), indicating its role as a bridge between different topics like those about government relief measures. The clustering coefficient for “money” (~0.771) showed potential economic fallouts being a common concern among community members. “Job” had a high clustering coefficient (1.0) and relatively high closeness centrality (~0.0035), suggesting that discussions about job security and employment were highly focused within the network.

Users expressed a desire for non-partisan approaches to community issues, which is aligned with semantic network results. The relatively high eigenvector centralities for “care” and “community” (both ~0.655) and clustering coefficients (both 1.0). Such scores indicated how highly clustered and their connection to other well-connected nodes. Still, the terms “community” and “care” were among the lowest in terms of betweenness centrality scores (both 0.0), indicating such terms do not lie on the shortest path between other nodes outside their immediate clusters. That is, “community” and “care” did not bridge other parts of the network. This can be due to some members pushing for the separation between local and national political values when it comes to disaster recovery (Topic 16).

### 4.2 Unraveling the interconnections of knotted tensions (RQ2)

**Figure 4:**
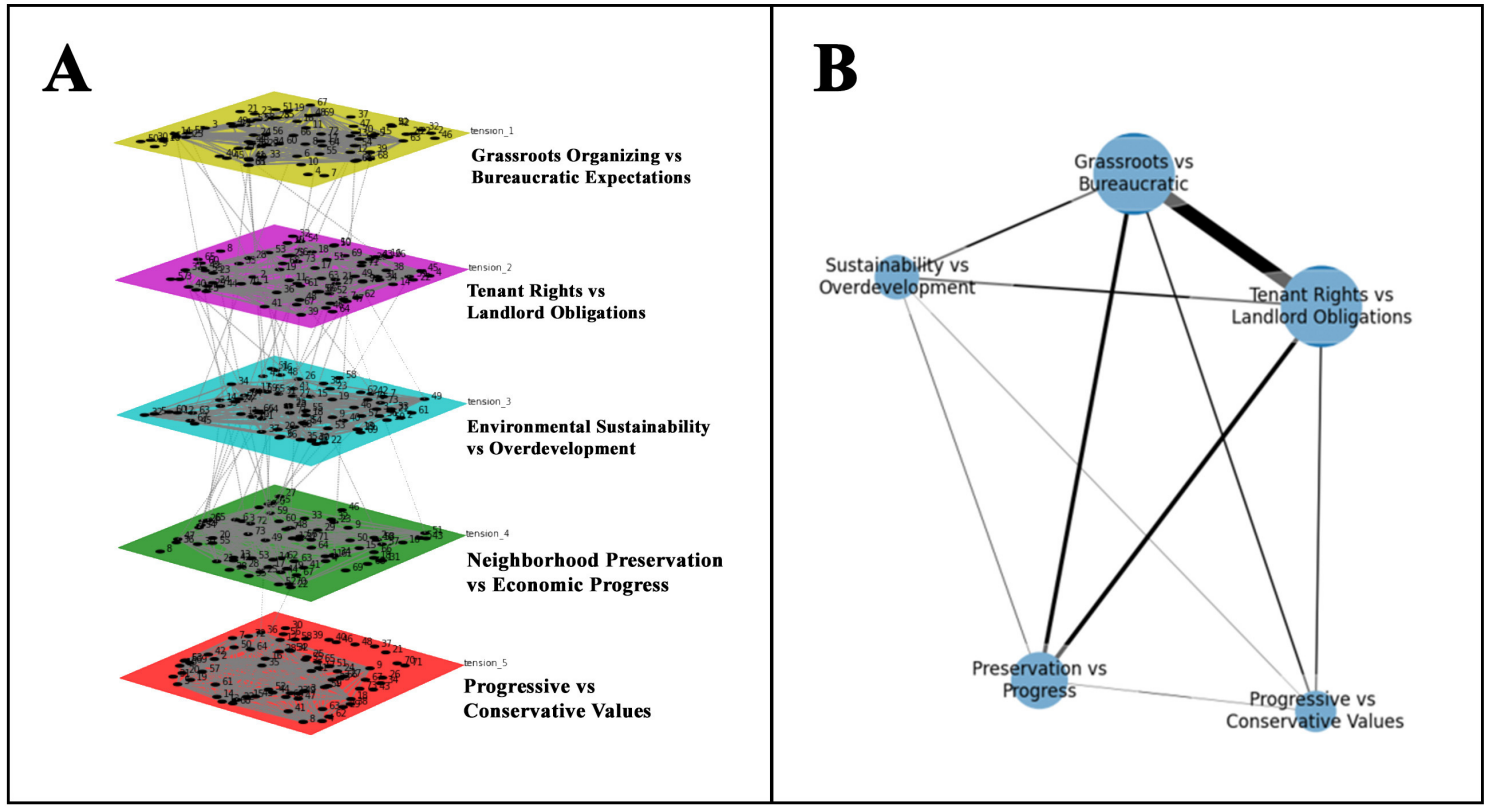
Multilayer Network and Bipartite Projection: A multilayer network showing connections between tensions **(A)** and a bipartite projection of the word-tension network **(B)**.

With this deep understanding of the tensions in this community, we next examined interconnections between tensions (RQ2). Figure 4 reflects these interconnections through both a multilayer visualization (panel A) and bipartite projection (panel B). The five layers in panel A reflect tensions raised across threads. Nodes represented words that were knotted within and across layers. These words formed intralayer edges, operationalized as the co-occurrence of words within conversation threads. Coupling edges reflected interconnections across layers of tensions. Threads extended vertically across layers and were connected by knots of identical words between different tensions. Figure 4 illustrates the interwoven nature of community discourse as certain words were more relevant than others, threading through multiple layers. The multilayer network offered a detailed, three-dimensional representation of the interplay between different community tensions and their commonalities in the aftermath of disaster. The right side of Figure 4 collapses the threads between layers into a bipartite projection. This projection allowed us to assess the overall discourse dynamics by viewing relationships between tension pairs from a bird's-eye view. Thicker lines reflected stronger connections (greater number of shared words) whereas thinner lines represented fewer connections. We included these different network visualizations to consider both the micro- and macro-level relationships within community discourse.

In comparing the two network models, the bipartite projection flattens the multidimensional nature of semantic relationships into a compressed, two-dimensional visual. In contrast, the multilayer model provides a richer representation of semantic connections by allowing words to nest within layers of discourse. Additionally, the multilayer model allows us to calculate interlayer correlations which quantifies the similarity between layers based on the presence of shared words. In the below section, we provide an in-depth look at the similarity between two layers which is not possible with the bipartite projection. The interlayer correlations from the multilayer model, however, helps to maintain the integrity and distinctiveness of discourse themes while simultaneously measuring their word-level similarities.

#### 4.2.1 Layer 1 and layer 5: the political undercurrents of crisis response

**Figure 5:**
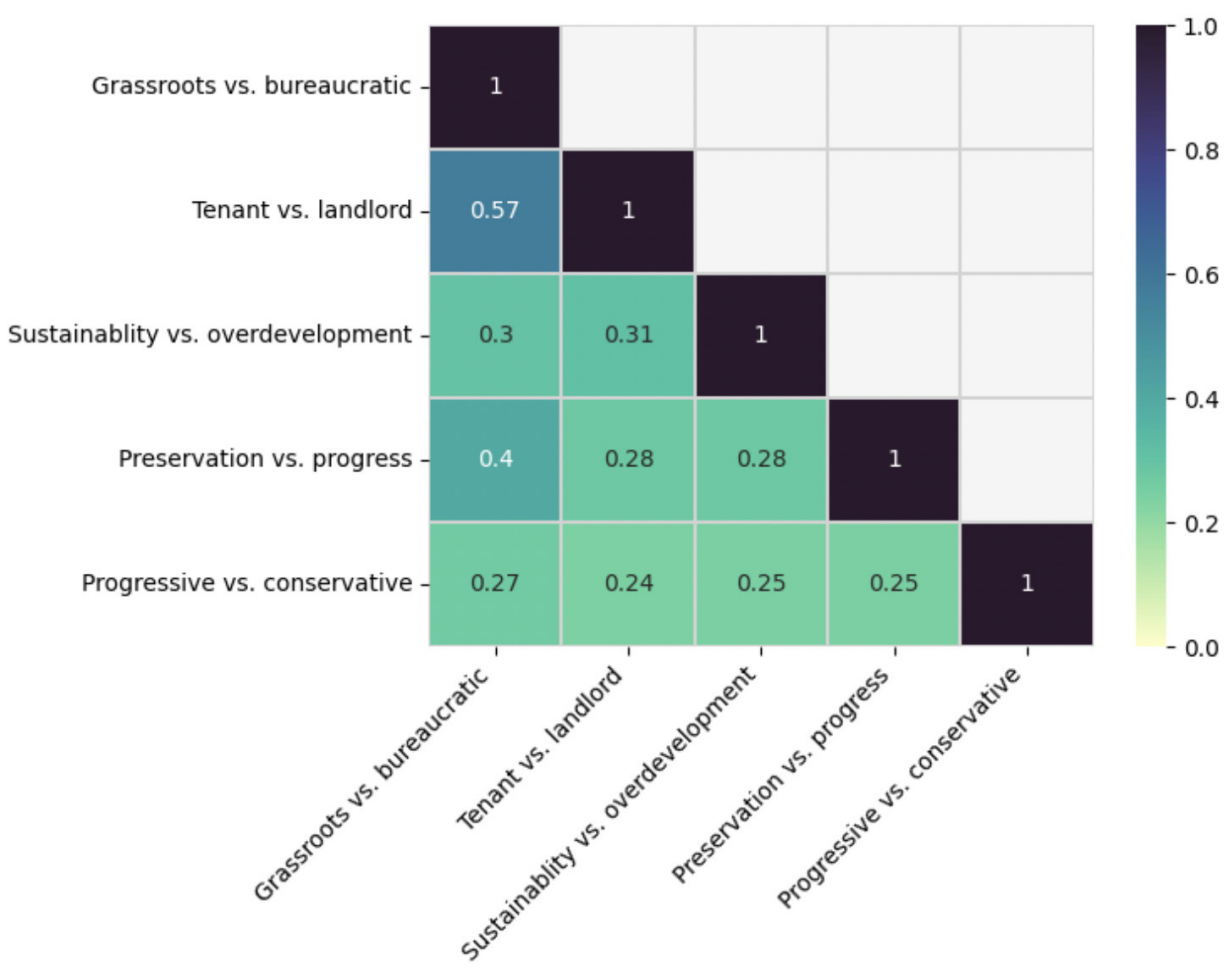
Interlayer Correlations: This heatmap represents the interlayer correlations of the coupling edges across layers in the multilayer network. Each square contains the correlation coefficient between two different tensions. Coefficients range from 0 (no correlation) to 1 (perfect correlation).

Tension 5 (progressive vs. conservative values) was the least correlated across all tensions (Figure 5), sharing strongest similarity with Tension 1 (grassroots organizing vs. bureaucratic expectations). While not the highest interlayer correlation score (J ~0.26), the similarity between both tensions suggested meaningful overlap in discourse. The unique words that formed coupling edges between Tensions 5 and 1 included terms like “administration,” “COVID-19,” “municipal,” “national,” “policy,” “president,” and “trump.” These words threaded the community's local discourse (lowercase) to broader Discourse (capitalized). Mentioning “trump” and “president” within the context of the 2020 presidential election suggested that discussions in Tension 5 resonated with the theme of bureaucratic expectations, particularly concerning frustrations over disaster response and preparedness in Tension 1. The presence of “COVID-19” pointed to the pandemic serving as a backdrop to these discussions, which involved debates over masks, transitioning to online instruction, and the COVID-19 testing shortage.

#### 4.2.2 Layer 1 and layer 2: community resilience in the face of adversity

Tension 1 (Grassroots Organizing vs. Bureaucratic Expectations) and Tension 2 (Tenant Rights vs. Landlord Obligations) shared the highest interlayer correlation (J ~0.56). The interconnection between these two tensions pointed to the entanglement of discourses in the aftermath of a crisis. These tensions share unique interlayer edges that include terms like “organize,” “utilities,” and “vulnerable.” These words acted as knots between these layers and spoke to the active engagement of members that organized to help their borough's most vulnerable residents. Shared interlayer edges of terms like “insurance,” “FEMA,” “rental,” “hotel,” “damage,” “rebuild,” and “health” highlighted the challenge of addressing disasters while also confronting systemic issues through advocacy, evidenced by “fight” and “protest.” These tensions also share interlayer edges between words that reflect local leaders like “mayor” and “council.” In Tension 1, community members called for the mayor and council to take responsibility for the lack of preparation and subsequent disaster response. Whereas, in Tension 2, users called for officials to support displaced community members who were being treated unfairly by a powerful landlord. When taken together, such words reflect the interconnections that represent the need for local leadership in community organizing efforts. This necessity for official support highlights the structural power dynamics that residents can face when confronting unfair housing practices. Still, the need to organize because leaders are not fulfilling expectations emphasizes the potential for public officials to also hinder community efforts.

#### 4.2.3 Layer 2 and layer 4: housing inequities and overdevelopment

Tension 2 (tenant rights vs. landlord obligations) and Tension 4 (neighborhood preservation vs. overdevelopment) had a lower layer correlation score (J ~ 0.27). Still, their unique interlayer edges offered a glimpse into the intersection between environmental concerns and existing housing issues. Shared knots like “broke,” “complaint,” “heat,” and “repair,” suggested issues with the maintenance whereas words like “mold” and “wet” pointed to environmental sustainability issues like water damage due to worsening flood zoning. The words “slumlord,” “lawyer,” and “legal” are unique to these tensions, which pointed to the need for legal recourse in both tension layers. Tensions 2 and 4 also shared the term “traumatic,” which suggested the emotional and psychological impact of these disasters on communities.

#### 4.2.4 Layer 1 and layer 4: grassroots organizing against environmental challenges

The knots between Tension 1 (grassroots organizing vs. bureaucratic expectations) and Tension 4 (environmental sustainability vs. overdevelopment) reflected the interconnection between community organizing around environmental issues, coupled with concerns about government action/inaction. These tensions had a relatively strong layer correlation (J ~0.39), indicating significant overlap in discourse. These tensions shared the unique word of “Sandy,” in reference to Superstorm Sandy, an extreme storm that caused catastrophic damage to the area in 2012. These tensions also shared words like “angry,” “lying,” “poor,” and “worry,” which pointed to similar expressions of emotion or sentiment in both these tensions. Terms such as “anger” and “worry,” along with “elected,” “failed,” and “respect” were in reference to government officials and their effectiveness. The word “failed” implied group members' dissatisfaction with the town council's response to environmental challenges. The term “respect” suggested an appeal for authorities to heed the voices and needs of the community.

## 5 Discussion

### 5.1 Overcoming information loss in bipartite networks

The goal of this work was to alleviate the information loss that comes with using typical bipartite projections (Opsahl, 2013; Everett and Borgatti, 2013; Yang and González-Bailón, 2016) and to quantify tensional knots (e.g., Sheep et al., 2017). The bipartite projection in this study offered a high-level view of the tension-to-tension connections between words that are uniquely shared. Although useful, this projection is unable to fully convey the multilayer network's depiction of the intricacy and hierarchical structure of language (Collins and Quillian, 1972; Rice and Danowski, 1993). This study illustrates the word structure buried in discourse topics through the use of a multilayer technique (Woelfel et al., 1980). Our findings illuminate the interconnections within and between community tensions that traditional bipartite networks cannot fully capture. For example, Layer 1 (Grassroots Organizing vs. Bureaucratic Expectations) showed overlap with Layer 5 (Progressive vs. Conservative Values). Their overlap provides evidence for the strength of a multilayer approach in revealing how discourse reflecting local issues can resonate with and can be shaped by larger socio-political conversations. Moreover, the thematic tensions between Layer 2 (Tenant Rights vs. Landlord Obligations) and Layer 1 (Grassroots Organizing vs. Bureaucratic Expectations) exhibit the highest interlayer correlation which, when depicted through the multilayer network structure, demonstrates the interconnection between local leadership and organizing efforts in the face of disaster. Collectively, this paper advocates for a multilayer approach that better models the semantic structure of interconnected tensions within public discourse.

### 5.2 A multilayer approach to modeling semantic networks

This study utilizes multilayer network methods to investigate semantic connections within and between layers of community discourse. We argue that more traditional bipartite approaches overlook how tensions are knotted (interconnected) within discourse, potentially oversimplifying interpretation. We find that shared terms across layers of discourse function as discursive resources (i.e., tools for dialogue and understanding; Hardy et al., 2005). This study argues that identifying discursive resources through multilayer network analysis has the potential to help practitioners navigate community tensions more effectively. Recall in the findings how the term “traumatic” appeared in discussions about poor housing conditions (Tension 2) and threats to neighborhood identity (Tension 4). Understanding how “traumatic” interconnects tension layers can help practitioners design messaging that addresses both housing and community preservation. For instance, “traumatic” in Tension 2 (Tenant Rights vs. Landlord Obligations) and Tension 4 (Neighborhood Preservation vs. Overdevelopment) suggests that inequitable access to safe housing can have implications for not only individual wellbeing but also community stability.

In this research, we demonstrate how interlayer correlations in multilayer semantic networks might offer a richer understanding of the interactions between various themes. Researchers can gain a deeper grasp of social media conversation in neighborhood situations by building a semantic network comprising several discourse layers and identifying the ways in which particular words or concepts serve as links between different topic layers.

This approach could also be used directly by community leaders or elected officials–allowing them to examine the themes and tensions that arise in public discourse across a range of settings. In the context of disaster response, this could help local governments, non-governmental organizations (NGOs), and community leaders more quickly identify and respond to community needs. Using a multilayer analysis can also enable leaders to develop intervention strategies that tackle the complexity of issues rather than approaching problems in isolation. This approach can help officials design multifaceted strategies that target the root of knotted tensions, saving them time and resources by minimizing redundant efforts.

Observing single semantic networks might oversimplify how discussion topics interact or evolve over time. We recommend that future work investigates how multilayer networks help yield a more accurate interpretation of semantic data when compared to single layer networks. Previous research also acknowledges how organizational initiatives for community improvement are often misaligned with what community members perceive as important issues (Zoller, 2000). Examining the interconnections in social media discourse is one way to address this alignment by providing a deeper understanding of community challenges.

### 5.3 Online community organizing

This study sheds light upon organizing practices and the role of community leaders in navigating local tensions. Our findings reveal that local leaders (e.g., mayor, town council) serve as both catalysts and barriers to managing local issues. This can be due to a lack of mutual understanding of issues, which can have implications for the success of community initiatives (Koschmann and Laster, 2011; Zoller, 2000). Also, disparities in power and knowledge between community leaders and organizational officials (Cooper, 2021) can make collaborations between communities and organizations more challenging (Gordon and Lopez, 2019). As previously discussed, community members demanded the mayor and town council put an end to illegal housing practices while simultaneously organizing themselves to participate in collective action. This finding illustrates a “both-and” strategy (Fairhurst and Putnam, 2024; Smith and Lewis, 2011) which has been found to help individuals navigate tensions (Berti and Simpson, 2021). Our data provides additional evidence for the “both-and” strategy as users expressed how to enact change (e.g., take legal action, attend town hall meetings) while still acknowledging power differentials and thus the need for elected officials. Future research should investigate strategies to enhance the efficacy of community organizing under varying bureaucratic conditions and also measure their impact on policy changes.

This study builds upon prior work by demonstrating the need for a coordinated approach that considers the interconnected nature of community issues. Community members' organizing activities included advocating for displaced low-income residents with a plea to the mayor and town council to address the town's housing issues. Theoretically, we recommend that future work investigates the efficacy of different strategies (e.g., the both-and strategy; Fairhurst and Putnam, 2023; Smith and Lewis, 2011) in community organizing contexts under different bureaucratic conditions. Practically, we encourage policymakers and organizational leaders to recognize the importance of engaging with and empowering community voices, when designing interventions to strengthen disaster resilience.

## 6 Limitations and conclusion

Like any research, the decisions made to formulate this research generate their own cost-benefit tensions. The decision to treat the data as cross-sectional over months of discussions did not capture the nature of conversations- that they unfold over time- or of post-disaster response and recovery, which are dynamic, compressing and expanding time for the victims and institutions involved. Future research should consider a longitudinal analysis to fully understand how these tensions evolve over time (Chewning et al., 2024). Tracking the discussions across multiple phases of disaster recovery could enable researchers to observe how the prominence and nature of tensions shift in response to changing community needs. By analyzing the data cross-sectionally, however, this paper accomplished an extension of paradoxical tensions theory in terms of how tensions are connected by common language. This study also illustrated the use of multilayer semantic networks as an additional method to help reveal the structures that generate meaning. Additionally, while we believe our multilayer semantic network approach allows for more nuanced understanding of community discourse, there may be settings in which this approach is not applicable. Our study focused on a single neighborhood forum–thus the ties between tensions meaningfully reflect connections, or lack thereof, in this set of public discourse. In settings where data reflects discussion among disparate publics, these across-layer connections may become less meaningful as different publics may be grappling with different layers of tension.

## Figures and Tables

**Figure A1:**
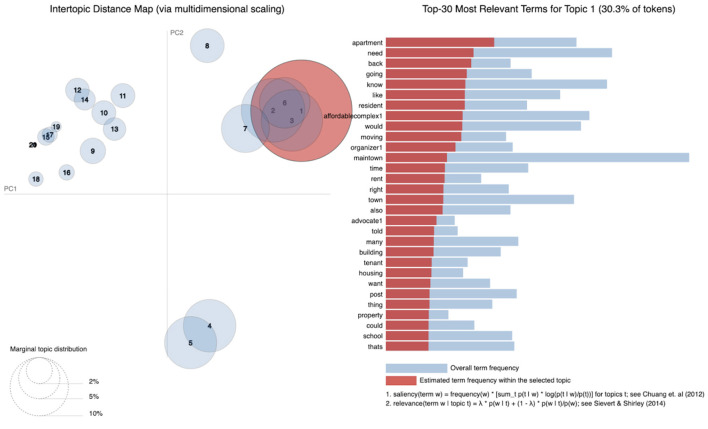
(Intertopic Distance Map): The Intertopic Distance Map shows the relationships between topics in a two-dimensional space using multidimensional scaling. Each circle represents a topic, and the size of the circle indicates the relative frequency of that topic in the dataset. The distances between circles indicate the similarity of topics; closer topics have more overlapping terms, while distant topics are more distinct in their word usage. The right panel shows the Top-30 Most Relevant Terms for Topic 1, accounting for 30.3% of the tokens in the dataset.

## Data Availability

The raw data supporting the conclusions of this article will be made available by the authors, without undue reservation.

## Footnotes

^1^r/TheRedPill is a subreddit focused on men's rights.

^2^Reminiscent of Mintz and Wayne (2016) polythink decision-making dynamic which occurs when there is a plurality of opinion (i.e., large number of differing views).
